# Assessment of Disparities Associated With a Crisis Standards of Care Resource Allocation Algorithm for Patients in 2 US Hospitals During the COVID-19 Pandemic

**DOI:** 10.1001/jamanetworkopen.2021.4149

**Published:** 2021-03-19

**Authors:** Hayley B. Gershengorn, Gregory E. Holt, Andrew Rezk, Stefanie Delgado, Nayna Shah, Arshia Arora, Leah B. Colucci, Belen Mora, Rahul S. Iyengar, Andy Lopez, Bianca M. Martinez, Joseph West, Kenneth W. Goodman, Daniel H. Kett, Jeffrey P. Brosco

**Affiliations:** 1 Division of Pulmonary, Critical Care, and Sleep Medicine, Department of Medicine, University of Miami Miller School of Medicine, Miami, Florida; 2 Division of Critical Care Medicine, Albert Einstein College of Medicine, Bronx, New York; 3 University of Miami Miller School of Medicine, Miami, Florida; 4 Department of Public Health Sciences, University of Miami Miller School of Medicine, Miami, Florida; 5 Institute for Bioethics and Health Policy, University of Miami Miller School of Medicine, Miami, Florida; 6 Department of Pediatrics, University of Miami Miller School of Medicine, Miami, Florida

## Abstract

**Importance:**

Significant concern has been raised that crisis standards of care policies aimed at guiding resource allocation may be biased against people based on race/ethnicity.

**Objective:**

To evaluate whether unanticipated disparities by race or ethnicity arise from a single institution’s resource allocation policy.

**Design, Setting, and Participants:**

This cohort study included adults (aged ≥18 years) who were cared for on a coronavirus disease 2019 (COVID-19) ward or in a monitored unit requiring invasive or noninvasive ventilation or high-flow nasal cannula between May 26 and July 14, 2020, at 2 academic hospitals in Miami, Florida.

**Exposures:**

Race (ie, White, Black, Asian, multiracial) and ethnicity (ie, non-Hispanic, Hispanic).

**Main Outcomes and Measures:**

The primary outcome was based on a resource allocation priority score (range, 1-8, with 1 indicating highest and 8 indicating lowest priority) that was assigned daily based on both estimated short-term (using Sequential Organ Failure Assessment score) and longer-term (using comorbidities) mortality. There were 2 coprimary outcomes: maximum and minimum score for each patient over all eligible patient-days. Standard summary statistics were used to describe the cohort, and multivariable Poisson regression was used to identify associations of race and ethnicity with each outcome.

**Results:**

The cohort consisted of 5613 patient-days of data from 1127 patients (median [interquartile range {IQR}] age, 62.7 [51.7-73.7]; 607 [53.9%] men). Of these, 711 (63.1%) were White patients, 323 (28.7%) were Black patients, 8 (0.7%) were Asian patients, and 31 (2.8%) were multiracial patients; 480 (42.6%) were non-Hispanic patients, and 611 (54.2%) were Hispanic patients. The median (IQR) maximum priority score for the cohort was 3 (1-4); the median (IQR) minimum score was 2 (1-3). After adjustment, there was no association of race with maximum priority score using White patients as the reference group (Black patients: incidence rate ratio [IRR], 1.00; 95% CI, 0.89-1.12; Asian patients: IRR, 0.95; 95% CI. 0.62-1.45; multiracial patients: IRR, 0.93; 95% CI, 0.72-1.19) or of ethnicity using non-Hispanic patients as the reference group (Hispanic patients: IRR, 0.98; 95% CI, 0.88-1.10); similarly, no association was found with minimum score for race, again with White patients as the reference group (Black patients: IRR, 1.01; 95% CI, 0.90-1.14; Asian patients: IRR, 0.96; 95% CI, 0.62-1.49; multiracial patients: IRR, 0.81; 95% CI, 0.61-1.07) or ethnicity, again with non-Hispanic patients as the reference group (Hispanic patients: IRR, 1.00; 95% CI, 0.89-1.13).

**Conclusions and Relevance:**

In this cohort study of adult patients admitted to a COVID-19 unit at 2 US hospitals, there was no association of race or ethnicity with the priority score underpinning the resource allocation policy. Despite this finding, any policy to guide altered standards of care during a crisis should be monitored to ensure equitable distribution of resources.

## Key Points

**Question:**

Is there an association of race and/or ethnicity with priority scores based on both short-term and longer-term estimated mortality used for resource allocation under crisis standards of care?

**Findings:**

In this retrospective cohort study of 1127 patients with 5613 patient-days in 2 US hospitals, there was no significant association of race or ethnicity with priority score.

**Meaning:**

In this study, the use of a crisis standards of care resource allocation policy based on both short-term and longer-term estimated mortality did not appear to discriminate against hospitalized patients based on self-identified race or ethnicity.

## Teaser

This cohort study evaluates whether unanticipated disparities by race or ethnicity would arise from the use of a resource allocation policy during the coronavirus disease 2019 (COVID-19) pandemic.

## Introduction

Crisis standards of care (CSC) are necessary to allow for equitable and transparent allocation of limited resources during times of excess demand.^1,2^ The coronavirus disease 2019 (COVID-19) pandemic has forced health care systems to confront the very real possibility that need for certain lifesaving resources (eg, intensive care unit [ICU] beds, ventilators, dialysis machines) may exceed supply. In response, regional governments^3^ and individual health care institutions^4^ revamped and, in some instances, de novo created CSC policies to aid in fair resource deployment.

While health care workers and lay people largely agree that triage following the default system of treating individuals on a first-come, first-served basis is not desirable,^5,6^ there remains significant disagreement about how, exactly, scarce resource allocation should occur. Clinicians tend to favor policies aimed at prioritizing those who will likely both survive the current illness (ie, short-term prognosis) and live longer following recovery (ie, longer-term prognosis).^5^ Conversely, the general public favors aiming to save the most lives^6^ while also considering acute illness prognosis (either prioritizing those most likely to die without^6^ or survive with^5^ treatment) without a focus on longer-term prognosis. Most regional and institutional CSC policies incorporate some measure of estimated short-term survival (eg, based on Sequential Organ Failure Assessment [SOFA] scores^7^), and many, although not all, also include an assessment of likely longer-term prognosis (eg, based on comorbidities).^3,4^

Significant concern has been raised that CSC policies—especially those that consider longer-term prognosis in triage scoring—may systematically deprioritize patients from underrepresented minority groups given the higher incidence of comorbidities among these populations resulting from systemic racism.^3,8,9^ In fact, compared with White lay people, Black individuals were significantly more likely to prefer a triage algorithm based on the principle of first come, first served and less likely to prefer one aimed at saving the most life-years,^5^ which may be a reflection of this very real concern.

In this study, we sought to evaluate whether our institution’s CSC policy, which is based on both short-term and longer-term prognosis, would result in unintended deprioritization of patients from minority groups during COVID-19. Given that our algorithm groups short-term prognosis into broader groups and assigns longer-term prognosis scores based on the presence of 1 or more comorbidities, we hypothesized that race- and ethnicity-related differences would be minimized and no unintended disparities would result.

## Methods

Data were collected as part of a quality improvement (QI) project aimed at evaluating the feasibility of implementing our newly created CSC policy, which depended on calculating daily priority scores for all patients at risk of mechanical ventilator triage due to surges in COVID-19 infection. We then conducted a retrospective cohort study of this data set. Data were collected daily from May 16 through July 14, 2020 from a midsize tertiary care hospital (May 26 through July 13, excluding May 31, June 20, and July 11) and a large quaternary care public hospital (June 30 through July 14, excluding July 6) at which University of Miami faculty attend.

Institutional review board approval was obtained from the University of Miami with a waiver of informed consent due to minimal risk to participants. The reporting of this work is consistent with the Strengthening the Reporting of Observational Studies in Epidemiology (STROBE) reporting guideline.^10^

### Institutional CSC Policy

**Figure 1.**
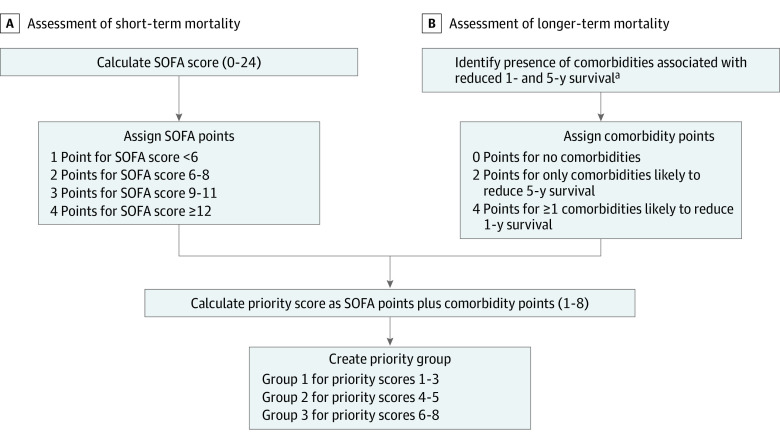
Crisis Standards of Care Resource Allocation Triage Point Scoring Algorithm SOFA indicates Sequential Organ Failure Assessment. ^a^Comorbidities expected to reduce 5-year survival included moderate dementia, malignancy with less than 10-year survival, New York Heart Association class III heart failure, moderate lung disease, end-stage kidney disease, and severe (ie, inoperable) coronary artery disease. Comorbidities expected to reduce 1-year survival included severe dementia, metastatic or stage IV cancer, New York Heart Association class IV heart failure, severe lung disease, cirrhosis with Model for End-Stage Liver Disease score greater than 20, traumatic brain injury with best Glasgow Coma Score motor response of 1, severe burns, cardiac arrest (unwitnessed, recurrent, or trauma-related), and severe immunocompromised states.

A team of 2 medical ethicists (K.W.G. and J.P.B) and 3 pulmonary and critical care physicians (G.E.H., D.H.K., and H.B.G.) met over videoconferencing in March and April 2020 to refine a CSC policy that had been created (but not finalized) in preparation for Ebola virus disease in 2014. The portion of our CSC policy aimed at resource allocation was designed to mirror those publicly available across multiple states and to align with guidance from experts.^3,4^ We had 3 primary goals in creating our policy: (1) to be fair and equitable; (2) to be actionable; and (3) to allocate scarce resources to those with both the greatest chance of surviving COVID-19 infection and living the longest. To this end, we created a primary allocation schema based on priority scores (1, indicating highest priority, through 8, indicating lowest priority) that were further consolidated into priority groups (1, indicating highest priority, through 3, indicating lowest priority) (Figure 1). Priority scores were a sum of points based on the likelihood of short-term mortality (based on daily SOFA score and categorized as 1-4 points, with 1 indicating a SOFA score of <6; 2, SOFA score 6-8; 3, SOFA score 9-11; 4, SOFA score ≥12) and longer-term mortality (based on comorbidities documented in the medical record, categorized as 0, 2, or 4 points). Points associated with comorbidities were assigned based on the likelihood of reduced 1-year (4 points) or 5-year (2 points) survival (eTable 1 in the Supplement). Patients only received 1 allotment of comorbidity points based the highest point value appropriate without a sum of scores from multiple comorbidities (ie, someone with 2 comorbidities with reduced 5-year survival and 3 comorbidities with reduced 1-year survival received 4 points for having at least 1 comorbidity that reduced 1-year survival). If needed, resource allocation would be based on priority groups (1-3) with ties within groups broken by comorbidities known to affect short-term recovery, then age (ie, younger patients receiving priority), followed by provision of an essential function within health care, then actual priority score (1-8), and, finally, lottery. If we were ever to implement this process, all allocation decisions would be made by a triage team consisting of the chief medical and nursing officers or designees, a critical care physician, an ethicist, and 1 person each from nursing or social work leadership. We recommended consideration be given to including a person with a disability and a member of the clergy. While our policy is not publicly available, it is the basis for a policy approved by the Florida Bioethics Network, the Florida Developmental Disability Council, and the Florida Hospital Association.^11^

### QI Project

To prepare for possible resource allocation need, we aimed to assess the feasibility of rapidly calculating daily priority scores for all patients at risk of potential ventilator allocation (to or away from such support). A team of 9 third- and fourth-year medical students were recruited and trained on how to calculate SOFA scores and how to review the electronic medical record for evidence of relevant comorbidities. A scoring how-to guide was created to enhance the likelihood that all students collected data similarly (eFigure 1 in the Supplement). Each day, students calculated scores for all relevant patients (each patient’s daily score was calculated by a single student) and entered them into a daily log in Excel 2013 (Microsoft Corp), which was kept on a secured Health Insurance Portability and Accountability Act–protected cloud-based server. At the tertiary hospital, the SOFA scores were automatically calculated by the electronic health record using an algorithm built locally and validated with medical record review prior to use. At the quaternary hospital, SOFA scores were calculated manually by students.

### Study Cohort

We included all patients entered into the QI data set in the cohort study. Patients were selected for inclusion in the QI project if they were admitted to an adult COVID-19 unit (ICU and non-ICU) at either hospital. At the tertiary hospital, we also included patients without COVID-19 who were admitted to an ICU or intermediate care unit and who were currently receiving mechanical ventilation (invasive or noninvasive) or high-flow nasal cannula considering that any ventilator allocation would apply to patients with and without COVID-19; patients outside COVID-19 units were not included at the quaternary hospital due to QI project–related resource limitations. We excluded patient-days without available SOFA scores and patients without comorbidity data (eFigure 2 in the Supplement). All analyses were done with the observation at the level of the patient (not patient-day).

### Exposures

We considered race (ie, White, Black, Asian, or multiracial) and ethnicity (Non-Hispanic or Hispanic) as separate exposures. Both exposures were taken from information provided in the electronic medical record, which is based on patient self-identification (or surrogate input if patients were not able).

### Outcomes

We evaluated 2 coprimary outcomes (ie, maximum and minimum priority score [1-8]) for each patient across all available patient-days of data. We chose the priority score (rather than priority group) to allow for a more granular analysis and because these scores would be used to break ties within priority groups. We considered the maximum and minimum score from each patient’s daily scores across the study period because the high or low scores for each patient would be likely to determine whether they would be denied (maximum score) or would receive (minimum score) access to resources. Secondary outcomes included both maximum and minimum priority groups, SOFA scores, and SOFA points. How tiebreakers (eg, comorbidities affecting short-term recovery, age, essential worker status) would affect triage was not evaluated.

### Statistical Analysis

We described the cohort using standard summary statistics and compared characteristics across groups using χ^2^ and Kruskal-Wallis testing, as appropriate. To assess the independent associations of race and ethnicity with triage priority, we created a series of 8 multivariable Poisson regression models, 1 for each outcome (eg, maximum priority score). Potential confounders for both exposures were considered similar and were all included in each model, as follows: sex (male or female), preferred language (English, Spanish, or other), median income of home zip code (<$25 000, $25 000 to <$50 000, $50 000 to <$75 000, or ≥$75 000), primary insurer (Medicare/Medicaid, commercial, or none), age, admission to a COVID-19 ward, and hospital (tertiary or quaternary). Because each exposure (race and ethnicity) was considered a potential confounder for the other, a single model including both exposures was constructed to assess each exposure’s association with each outcome. Our primary models included complete cases; we conducted a sensitivity analysis including all patients and including an unknown category for all covariates. We conducted a second pair of sensitivity analyses excluding covariates that may track with race or ethnicity and are actually components of structural racism (ie, median income and primary insurance).

To determine whether including information about longer-term prognosis (ie, comorbidity points) was associated with the prioritization of patients of different races and ethnicities, we compared patient prioritization by SOFA points alone (categorized in 3 groups) vs priority groups (based on SOFA points plus comorbidities). Quantification of the association of including comorbidities was assessed as the proportion of patients in each race and ethnicity group who achieved higher or lower priority after comorbidity inclusion.

All statistical analyses were performed using Stata 16 (StataCorp) and Excel 2013 (Microsoft Corp). A 2-tailed *P* < .05 was considered statistically significant; no adjustment was made for multiple comparisons.

## Results

The cohort was composed of 1127 patients (675 [59.9%] from the tertiary hospital; median [interquartile range {IQR}] age, 62.7 [51.7-73.7]; 607 [53.9%] men) and 5613 days of data (3296 [58.7%] from the tertiary hospital). Overall, 711 (63.1%) were White patients, 323 (28.7%) were Black patients, 8 (0.7%) were Asian patients, 31 (2.8%) were multiracial patients, and in 54 patients (4.8%), race was unknown; 480 (42.6%) were Non-Hispanic patients, 611 (54.2%) were Hispanic patients, and 36 (3.2%) had unknown ethnicity.

**Figure 2.**
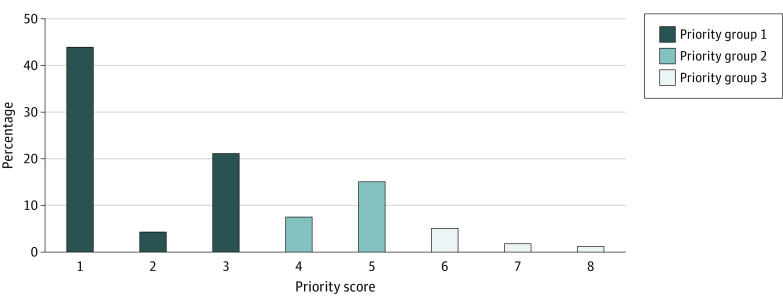
Distribution of Maximum Priority Scores Across Cohort

A total of 782 patients (69.4%) had a maximum priority group assignment of 1, while 255 (22.6%) were in group 2, and 90 (8.0%) were in group 3 (Table 1 and Figure 2). The median (IQR) maximum priority score for the cohort was 3 (1-4); the median (IQR) minimum score was 2 (1-3). Patients in maximum priority group 3 were more likely to be older (median [IQR] age: group 3, 68.5 [55.0-79.0] years; group 2, 66.3 [57.1-75.8] years; group 1, 61.0 [50.1-70.9] years; *P* < .001) with more comorbidities (those with reduced 5-year survival: group 3, 55 [61.1%]; group 2, 147 [57.6%]; group 1, 206 [26.3%]; *P* < .001; those with reduced 1-year survival: group 3, 70 [77.8%]; group 2, 147 [57.6%]; group 1, 0; *P* < .001). Patients with a maximum priority group of 3 were less likely to be admitted to a COVID-19 ward (group 3, 36 [40.0%]; group 2, 113 [44.3%]; group 1, 541 [69.2%]; *P* < .001); however, patients being cared for in a COVID-19 ward may have been admitted to general medical units while patients not receiving care in a COVID-19 ward were only admitted to ICUs or intermediate care units. Similar associations were found with minimum priority groups (eTable 2 in the Supplement).

**Table 1.** Baseline Characteristics of Cohort by Maximum Priority Group

Characteristic	Patients by priority group, No. (%)				*P* value	
	Full cohort (N = 1127)	1 (n = 782)	2 (n = 255)	3 (n = 90)	All groups	Group 3 vs 1
Days of data per patient, median (IQR), No.	3 (2-7)	3 (1-6)	3 (2-7)	6 (3-10)	<.001	<.001
Age, median (IQR), y^a^	62.7 (51.7-73.7)	61.0 (50.1-70.9)	66.3 (57.1-75.8)	68.5 (55.0-79.0)	<.001	<.001
Comorbidities^b^						
Reduce 5-y survival	408 (36.2)	206 (26.3)	147 (57.6)	55 (61.1)	<.001	<.001
Reduce 1-y survival	217 (19.3)	0	147 (57.6)	70 (77.8)	<.001	<.001
Race						
White	711 (63.1)	500 (63.9)	156 (61.2)	55 (61.1)	.25	.18
Black	323 (28.7)	227 (29.0)	71 (27.8)	25 (27.8)		
Asian	8 (0.7)	5 (0.6)	3 (1.2)	0		
Multiracial	31 (2.8)	22 (2.8)	7 (2.7)	2 (2.2)		
Unknown	54 (4.8)	28 (3.6)	18 (7.1)	8 (8.9)		
Ethnicity						
Non-Hispanic	480 (42.6)	319 (40.8)	119 (46.7)	42 (46.7)	.22	.18
Hispanic	611 (54.2)	440 (56.3)	128 (50.2)	43 (47.8)		
Unknown	36 (3.2)	23 (2.9)	8 (3.1)	5 (5.6)		
Sex						
Men	607 (53.9)	415 (53.1)	139 (54.5)	54 (60.0)	.07	.01
Women	509 (45.2)	362 (46.3)	113 (44.3)	33 (36.7)		
Neither or unknown	11 (1.0)	5 (0.6)	3 (1.2)	3 (3.3)		
Preferred language						
English	591 (52.4)	398 (50.9)	140 (54.9)	53 (58.9)	.34	.16
Spanish	489 (43.4)	351 (44.9)	104 (40.8)	34 (37.8)		
Other	35 (3.1)	27 (3.5)	7 (2.7)	1 (1.1)		
Unknown	12 (1.1)	6 (0.8)	4 (1.6)	2 (2.2)		
Primary insurance						
Medicare or Medicaid	360 (31.9)	219 (28.0)	95 (37.3)	46 (51.1)	<.001	<.001
Commercial	589 (52.3)	411 (52.6)	138 (54.1)	40 (44.4)		
None	153 (13.6)	134 (17.1)	16 (6.3)	3 (3.3)		
Unknown	25 (2.2)	18 (2.3)	6 (2.4)	1 (1.1)		
Median annual income for zip code^c^						
<$25 000	219 (19.4)	158 (20.2)	44 (17.3)	17 (18.9)	.60	.64
$25 000 to <$50 000	546 (48.4)	379 (48.5)	118 (46.3)	49 (54.4)		
$50 000 to <$75 000	236 (20.9)	154 (19.7)	64 (25.1)	18 (20.0)		
≥$75 000	71 (6.3)	51 (6.5)	17 (6.7)	3 (3.3)		
Unknown	55 (4.9)	40 (5.1)	12 (4.7)	3 (3.3)		
Receiving care in COVID-19 unit^d^	690 (61.2)	541 (69.2)	113 (44.3)	36 (40.0)	<.001	<.001

Abbreviation: IQR, interquartile range.

^a^ Age used is age on June 1, 2020; data missing for 3 (0.3%) patients; median (IQR) age varied by race (White patients, 63.3 [53.1-74.3] years; Black patients: 60.9 [48.7-71.2] years; Asian patients: 66.6 [47.6-70.7] years, multiracial patients: 66.1 [55.2-78.5] years, patients with unknown race: 64.1 [49.5-75.0] years; *P* = .02) but not ethnicity (non-Hispanic patients: 62.7 [51.7-71.9] years; Hispanic patients: 62.9 [52.3-75.4] years, patients with unknown ethnicity: 60.3 [42.3-75.0] years; *P* = .28).

^b^ Presence of at least 1 comorbidity likely to reduce 1-year and/or 5-year survival are both listed here; in assignment of points for priority scoring, patients with both categories of comorbidity were only allocated points for the more severe (ie, 1-year) comorbidity burden.

^c^ Data missing for 55 patients (4.9%).

^d^ COVID-19 status for each patient was not accessed; rather, this value indicates whether a patient was admitted to a ward serving patients with COVID-19 because these patients were segregated from those without COVID-19 by ward in both hospitals.

### Association of Race and Ethnicity With Triage Priority

There were no significant differences in maximum priority group across races (White patients: group 1, 500 [63.9%]; group 2, 156 [61.2%]; group 3, 55 [61.1%]; Black patients: group 1, 227 [29.0%]; group 2, 71 [27.8%]; group 3, 25 [27.8%]; *P* = .25) or ethnicities (Hispanic patients: group 1, 440 [56.3%]; group 2, 128 [50.2%]; group 3, 43 [47.8%]; *P* = .22). Similarly, no significant differences were found in race and ethnicity breakdowns across minimum priority groups.

After multivariable adjustment, there was no association of race with maximum priority score using White patients as the reference group (Black patients: incidence rate ratio [IRR], 1.00; 95% CI, 0.89-1.12; Asian patients: IRR, 0.95; 95% CI, 0.62-1.45; multiracial patients: IRR, 0.93; 95% CI, 0.72-1.19) or ethnicity using non-Hispanic patients as the reference group (Hispanic patients: IRR, 0.98; 95% CI, 0.88-1.10) (Table 2). Similarly, no association was found with minimum priority score using the same reference racial and ethnic reference groups (Black patients: IRR, 1.01; 95% CI, 0.90-1.14; Asian patients: IRR, 0.96; 95% CI, 0.62-1.49; multiracial patients: IRR, 0.81; 95% CI, 0.61-1.07; Hispanic patients: IRR, 1.00; 95% CI, 0.89-1.13). The only association found between self-identified race or ethnicity across any secondary outcomes was for maximum SOFA score, for which multiracial patients (compared with White patients) were more likely to have a higher SOFA score (IRR, 1.33; 95% CI, 1.12-1.59; *P* = .001) (eTables 3-5 in the Supplement). In the sensitivity analyses using the full cohort and assigning missing data to an unknown category (eTable 6 in the Supplement) and removing socioeconomic factors as covariates (eTable 7 and eTable 8 in the Supplement), results were qualitatively the same.

**Table 2.** Adjusted Association of Race and Ethnicity With Maximum and Minimum Priority Scores

Characteristic	Maximum priority score		Minimum priority score	
	IRR (95% CI)	*P* value	IRR (95% CI)	*P* value
Race				
White	1 [Reference]	NA	1 [Reference]	NA
Black	1.00 (0.89-1.12)	.94	1.01 (0.90-1.14)	.83
Asian	0.95 (0.62-1.45)	.81	0.96 (0.62-1.49)	.86
Multiracial	0.93 (0.72-1.19)	.56	0.81 (0.61-1.07)	.14
Ethnicity				
Non-Hispanic	1 [Reference]	NA	1 [Reference]	NA
Hispanic	0.98 (0.88-1.10)	.76	1.00 (0.89-1.13)	.98
Sex				
Male	1 [Reference]	NA	1 [Reference]	NA
Female	0.93 (0.86-1.00)	.05	0.97 (0.89-1.05)	.39
Preferred language				
English	1 [Reference]	NA	1 [Reference]	NA
Spanish	0.95 (0.86-1.06)	.37	0.95 (0.85-1.07)	.41
Other	0.86 (0.69-1.08)	.20	0.87 (0.69-1.11)	.26
Median annual income for zip code, $				
<25 000	1 [Reference]	NA	1 [Reference]	NA
25 000 to <50 000	1.07 (0.97-1.19)	.17	1.10 (0.99-1.22)	.08
50 000 to <75 000	1.01 (0.89-1.14)	.88	1.03 (0.90-1.17)	.69
≥75 000	1.00 (0.84-1.20)	.97	1.10 (0.91-1.33)	.32
Primary insurance				
Medicare/Medicaid	1 [Reference]	NA	1 [Reference]	NA
Commercial	0.84 (0.77-0.91)	<.001	0.83 (0.76-0.91)	<.001
None	0.66 (0.57-0.76)	<.001	0.66 (0.56-0.77)	<.001
Age^a^	1.01 (1.00-1.01)	<.001	1.01 (1.00-1.01)	<.001
Receiving care in COVID-19 unit	0.70 (0.63-0.78)	<.001	0.69 (0.62-0.77)	<.001
Quaternary hospital	0.97 (0.87-1.09)	.62	0.97 (0.86-1.09)	.64

Abbreviations: IRR, incident rate ratio; NA, not applicable.

^a^ Age on June 1, 2020; modeled as a continuous variable.

**Figure 3.**
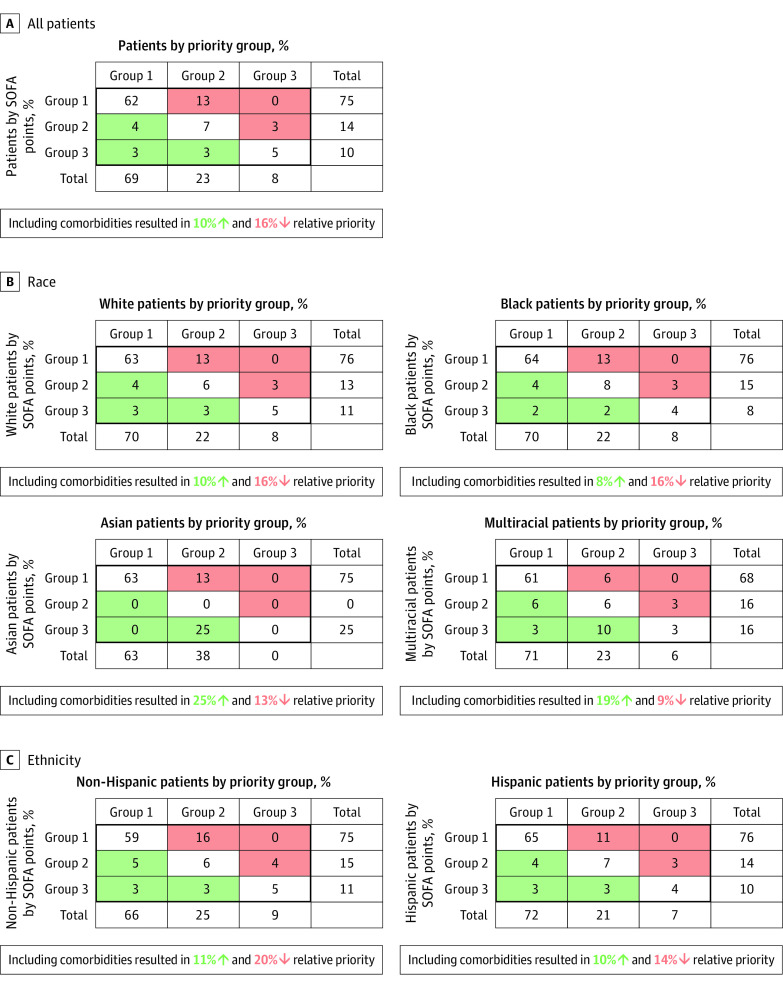
Comparison of Relative Triage Priority Based on Maximum Points With and Without Inclusion of Longer-Term Mortality Sequential Organ Failure Assessment (SOFA) points 3 and 4 combined in single group (group 3).

When comparing maximum priority group (based on SOFA plus comorbidity) information to triage groups based on maximum SOFA points alone, 10% of the cohort would receive higher and 16% lower priority for resource allocation with the inclusion of comorbidity data (Figure 3). This change in prioritization was similar for White patients (10% higher, 16% lower) and Black patients (8% higher, 16% lower). Asian patients (25% higher, 13% lower) and multiracial patients (19% higher, 9% lower) appeared to move into higher priority groups at greater rates than other groups with the inclusion of comorbidities. Inclusion of comorbidities resulted in Hispanic patients receiving higher prioritization 10% of the time (11% for non-Hispanic patients) and lower prioritization 14% of the time (20% for non-Hispanic patients). Comparable relative rates of reprioritization across races and ethnicities were seen when considering minimum priority group vs minimum SOFA point–based group (eFigure 3 in the Supplement).

## Discussion

As hypothesized, we found no association of race or ethnicity with either maximum or minimum priority score. Across 6 secondary outcomes, the only significant association identified was self-identification as a multiracial person (compared with White) with an increase in maximum SOFA score but not SOFA points. This finding is of no consequence for resource allocation because our CSC protocol used SOFA points, not SOFA score. Additionally, despite concerns that inclusion of comorbidity information would lead to deprioritization of individuals from underrepresented minority groups, the priority groups assigned to Black and White patients were similarly affected by the addition of comorbidity data. Asian and multiracial patients as well as those with Hispanic (vs non-Hispanic) ethnicity fared relatively better with the inclusion of comorbidity data.

There is good reason to be concerned that COVID-19–related CSC policies may negatively affect racial and ethnic minorities. Disparities have been identified in relation to COVID-19; test positivity rates, hospitalization, and, in some studies, mortality rates are higher among Black^12,13,14,15,16,17,18,19,20,21,22,23^ and Hispanic individuals.^13,14,15,16,22,23^ Moreover, prior work has demonstrated that seemingly race/ethnicity–agnostic scoring systems may disadvantage minority patients. e.g., Vigil et al^24,25^ found that being non-Hispanic Black or Hispanic (vs non-Hispanic White) was associated with being assigned a lower emergency severity index score on emergency department presentation.

There are several potential explanations for our findings that neither race nor ethnicity were associated with triage prioritization using our CSC policy. First, it is possible that there truly exists no association between race or ethnicity and triage priority when assigned using a composite of estimated short-term and longer-term survival. Evidence for higher comorbidity burdens among individuals from underrepresented minority groups is robust^26,27^ and has been the focus of many concerns regarding possible disparities related to CSC policies.^3,8,9^ There is also evidence that acuity of non–COVID-19 illness on ICU presentation^28^ and COVID-19–related lung involvement on hospital admission^29^ may be higher for individuals from racial/ethnic minority groups. However, our strategy of assigning a value only for the single most serious comorbidity a patient has and of grouping SOFA scores within broader buckets may have blunted some of these differences. It should be noted that the cohort included only patients after admission to a hospital. Race/ethnicity–associated differences in rates and timing of seeking hospital-based care and rates of hospital admission after presenting with COVID-19 may bias our findings. Second, our sample size may have been insufficient to identify a true association of race or ethnicity with triage priority. However, the relatively narrow confidence intervals surrounding the association of both Black (vs White) and Hispanic (vs non-Hispanic) patients with triage scoring strengthens our findings. Finally, our results may be affected by residual confounding, specifically socioeconomic factors. We used median income of a patient’s zip code and primary insurer to account partially for these influences, yet this adjustment is assuredly insufficient.

To our knowledge, ours is the first analysis to evaluate the association of race and ethnicity with a CSC policy during COVID-19. Its main strength stems from our diverse cohort, inclusive of more than 25% Black and more than 50% Hispanic patients. Additionally, this study allowed us to demonstrate that our scoring algorithm was successful in achieving score distribution across the cohort, a necessary step for any triage tool.

### Limitations

Our analysis has several limitations. First, longer-term survival was based on comorbidities identifiable from the electronic health record of each hospital. With differing access to care^30^ and potentially different hospital admission patterns, it is possible that comorbidities were underdiagnosed and, potentially, underdocumented for certain racial and ethnic subgroups. Moreover, medical students were tasked with abstracting comorbidity information, and their knowledge and experience may have affected accuracy. However, use of diagnoses available in the electronic health record simulates the process we would use in real-time were resource allocation triage needed. Second, assignment of a triage priority score is only the first step in the process of resource allocation. Factors that would be used in practice to break ties among patients in the same priority group were not considered; however, it is possible that inclusion of these factors might actually mitigate against bias because younger populations^31^ and health care workers^32^ are disproportionately from minority racial/ethnic groups. Moreover, ultimate triage decisions would be made by a separate triage team. Whether unintended bias would enter this latter portion of triage decision-making was not evaluated in our study; however, the separate triage team would be masked to patients’ race and ethnicity. Third, while Black and Hispanic patients were well represented in the cohort, we had few patients from other racial groups. Fourth, our study did not consider disability status because such information was not available at the time of data analysis. Fifth, the cohort consisted of patients admitted to 2 academic hospitals in Miami, a city with a diverse population and medical staff; the external generalizability of our findings to other settings is unknown. Additionally, the impact of collaboration between regional hospitals and triage across them was not considered. Similarly, our work may not be generalizable to health systems with different triage policies (eg, those that give lower priority to patients with greater numbers of comorbidities). Sixth, although neither hospital experienced a lack of access to ventilators, other aspects of care (eg, medication availability, admission of higher acuity patients to intermediate care units instead of ICUs) certainly deviated from standards of care during this time; whether this affected triage scoring is unknown but, unfortunately, reflects the reality of care during a crisis when such triage may be necessary. Seventh, race and ethnicity were obtained from the electronic health record; misclassification based on erroneous race or ethnicity assignment as well as the intrinsic challenges associated with asking people to self-identify into racial and ethnic categories may have introduced bias.^33^

## Conclusions

In this cohort study of adult patients admitted to a COVID-19 unit at 2 US hospitals, there was no association of race or ethnicity with the priority score underpinning a resource allocation policy. The COVID-19 pandemic is a stark reminder of how unfair our society can be. Racial and ethnic minority groups have endured a disproportionate brunt of the disease and its consequences in the United States. Clinicians, hospital administrators, and governmental leaders have an obligation to minimize, and not exacerbate, such disparities. At the same time, the need to employ CSC amid a global pandemic cannot be ignored. The findings of this study that such a policy, based on both short-term and longer-term expected survival, did not appear to unintentionally disadvantage patients from underrepresented minority groups is reassuring. However, in the event that any such policy is activated, ongoing vigilance for evidence of such disparities will be essential and should be included in the implementation of any CSC policy.

## Notes

Supplement. **eFigure 1.** Scoring How-To Guide **eFigure 2.** Study Flow Diagram **eTable 1.** Comorbidities Included in Triage Score **eTable 2.** Baseline Characteristics of Cohort by Minimum Priority Group **eTable 3.** Adjusted Association of Race and Ethnicity with Maximum and Minimum Priority Groups **eTable 4.** Adjusted Association of Race and Ethnicity with Maximum and Minimum Sequential Organ Failure Assessment Scores **eTable 5.** Adjusted Association of Race and Ethnicity with Maximum and Minimum Sequential Organ Failure Assessment Points **eTable 6.** Adjusted Association of Race and Ethnicity with Maximum and Minimum Priority Scores Including Patients with Missing Covariate Information **eTable 7.** Adjusted Association of Race and Ethnicity with Maximum and Minimum Priority Scores Without Median Income as a Model Covariate **eTable 8.** Adjusted Association of Race and Ethnicity with Maximum and Minimum Priority Scores Without Median Income or Primary Insurance as Model Covariates **eFigure 3.** Comparison of Relative Triage Priority Based on Minimum Points With and Without Inclusion of Longer-Term Mortality

## References

[1] Institute of Medicine Committee on Guidance for Establishing Standards of Care for Use in Disaster Situations. *Guidance for Establishing Crisis Standards of Care for Use in Disaster Situations: A Letter Report*. Altevogt BM, Stroud C, Hanson SL, Hanfling D, Gostin LO, eds. Published 2009. Accessed August 24, 2020. https://www.nap.edu/download/12749# 25032361

[2] American Medical Association. Crisis standards of care: guidance from the AMA Code of Medical Ethics. Updated April 5, 2020. Accessed August 24, 2020. https://www.ama-assn.org/delivering-care/ethics/crisis-standards-care-guidance-ama-code-medical-ethics

[3] Cleveland Manchanda EC, Sanky C, Appel JM. Crisis standards of care in the USA: a systematic review and implications for equity amidst COVID-19. J Racial Ethn Health Disparities. Published online August 13 2020. doi:10.1007/s40615-020-00840-5 32789816 PMC7425256

[4] White DB, Katz M, Luce J, . Allocation of scarce critical care resources during a public health emergency. University of Pittsburgh School of Medicine. Department of Critical Care Medicine. Published April 15, 2020. Accessed August 24, 2020. https://ccm.pitt.edu/sites/default/files/UnivPittsburgh_ModelHospitalResourcePolicy_2020_04_15.pdf

[5] Biddison ELD, Gwon HS, Schoch-Spana M, . Scarce resource allocation during disasters: a mixed-method community engagement study. Chest. 2018;153(1):187-195. doi:10.1016/j.chest.2017.08.001 28802695

[6] Grover S, McClelland A, Furnham A. Preferences for scarce medical resource allocation: differences between experts and the general public and implications for the COVID-19 pandemic. Br J Health Psychol. 2020;25(4):889-901. doi:10.1111/bjhp.12439 32562512 PMC7323072

[7] Vincent JL, Moreno R, Takala J, . The SOFA (Sepsis-related Organ Failure Assessment) score to describe organ dysfunction/failure: on behalf of the Working Group on Sepsis-Related Problems of the European Society of Intensive Care Medicine. Intensive Care Med. 1996;22(7):707-710. doi:10.1007/BF01709751 8844239

[8] Cleveland Manchanda E, Couillard C, Sivashanker K. Inequity in crisis standards of care. N Engl J Med. 2020;383(4):e16. doi:10.1056/NEJMp2011359 32402154

[9] Caraccio C, White RS, Jotwani R. No protocol and no liability: a call for COVID crisis guidelines that protect vulnerable populations. J Comp Eff Res. 2020;9(12):829-837. doi:10.2217/cer-2020-0090 32705880 PMC7379972

[10] von Elm E, Altman DG, Egger M, Pocock SJ, Gøtzsche PC, Vandenbroucke JP; STROBE Initiative. The Strengthening the Reporting of Observational Studies in Epidemiology (STROBE) statement: guidelines for reporting observational studies. Ann Intern Med. 2007;147(8):573-577. doi:10.7326/0003-4819-147-8-200710160-00010 17938396

[11] Florida Bioethics Network. Ethics guidelines for crisis standards of care in public health emergencies. Updated May 1, 2020. Accessed October 9, 2020. https://fbn.miami.edu/_assets/pdf/resources/covid-19-resources/csc-fbn-6.pdf

[12] Adegunsoye A, Ventura IB, Liarski VM. Association of black race with outcomes in COVID-19 disease: a retrospective cohort study. Ann Am Thorac Soc. 2020;17(10):1336-1339. doi:10.1513/AnnalsATS.202006-583RL 32643398 PMC7640625

[13] Adhikari S, Pantaleo NP, Feldman JM, Ogedegbe O, Thorpe L, Troxel AB. Assessment of community-level disparities in coronavirus disease 2019 (COVID-19) infections and deaths in large US metropolitan areas. JAMA Netw Open. 2020;3(7):e2016938. doi:10.1001/jamanetworkopen.2020.16938 32721027 PMC7388025

[14] Cowger TL, Davis BA, Etkins OS, . Comparison of weighted and unweighted population data to assess inequities in coronavirus disease 2019 deaths by race/ethnicity reported by the US Centers for Disease Control and Prevention. JAMA Netw Open. 2020;3(7):e2016933. doi:10.1001/jamanetworkopen.2020.16933 32721026 PMC7388022

[15] Holtgrave DR, Barranco MA, Tesoriero JM, Blog DS, Rosenberg ES. Assessing racial and ethnic disparities using a COVID-19 outcomes continuum for New York State. Ann Epidemiol. 2020;48:9-14. doi:10.1016/j.annepidem.2020.06.010 32723697 PMC7323653

[16] Hsu HE, Ashe EM, Silverstein M, . Race/ethnicity, underlying medical conditions, homelessness, and hospitalization status of adult patients with COVID-19 at an urban safety-net medical center—Boston, Massachusetts, 2020. MMWR Morb Mortal Wkly Rep. 2020;69(27):864-869. doi:10.15585/mmwr.mm6927a3 32644981 PMC7727597

[17] Jehi L, Ji X, Milinovich A, . Development and validation of a model for individualized prediction of hospitalization risk in 4,536 patients with COVID-19. PLoS One. 2020;15(8):e0237419. doi:10.1371/journal.pone.0237419 32780765 PMC7418996

[18] Killerby ME, Link-Gelles R, Haight SC, ; CDC COVID-19 Response Clinical Team. Characteristics associated with hospitalization among patients with COVID-19—metropolitan Atlanta, Georgia, March-April 2020. MMWR Morb Mortal Wkly Rep. 2020;69(25):790-794. doi:10.15585/mmwr.mm6925e1 32584797 PMC7316317

[19] Li AY, Hannah TC, Durbin JR, . Multivariate analysis of Black race and environmental temperature on COVID-19 in the US. Am J Med Sci. 2020;360(4):348-356. doi:10.1016/j.amjms.2020.06.015 32709397 PMC7305735

[20] Patel AP, Paranjpe MD, Kathiresan NP, Rivas MA, Khera AV. Race, socioeconomic deprivation, and hospitalization for COVID-19 in English participants of a national biobank. Int J Equity Health. 2020;19(1):114. doi:10.1186/s12939-020-01227-y 32631328 PMC7336098

[21] Poulson M, Geary A, Annesi C, . National disparities in COVID-19 outcomes between Black and White Americans. J Natl Med Assoc. Published August 7, 2020. doi:10.1016/j.jnma.2020.07.009 32778445 PMC7413663

[22] Rozenfeld Y, Beam J, Maier H, . A model of disparities: risk factors associated with COVID-19 infection. Int J Equity Health. 2020;19(1):126. doi:10.1186/s12939-020-01242-z 32727486 PMC7387879

[23] Vahidy FS, Nicolas JC, Meeks JR, . Racial and ethnic disparities in SARS-CoV-2 pandemic: analysis of a COVID-19 observational registry for a diverse US metropolitan population. BMJ Open. 2020;10(8):e039849. doi:10.1136/bmjopen-2020-039849 32784264 PMC7418666

[24] Vigil JM, Alcock J, Coulombe P, . Ethnic disparities in emergency severity index scores among U.S. Veteran’s Affairs emergency department patients. PLoS One. 2015;10(5):e0126792. doi:10.1371/journal.pone.0126792 26024515 PMC4449190

[25] Vigil JM, Coulombe P, Alcock J, . Patient ethnicity affects triage assessments and patient prioritization in U.S. Department of Veterans Affairs emergency departments. Medicine (Baltimore). 2016;95(14):e3191. doi:10.1097/MD.0000000000003191 27057847 PMC4998763

[26] Arasteh K. Prevalence of comorbidities and risks associated with COVID-19 among Black and Hispanic populations in New York City: an examination of the 2018 New York City Community Health Survey. J Racial Ethn Health Disparities. 2020;(Aug). doi:10.1007/s40615-020-00844-1 32794024 PMC7425794

[27] Balasubramanian BA, Garcia MP, Corley DA, . Racial/ethnic differences in obesity and comorbidities between safety-net and non–safety-net integrated health systems. Medicine (Baltimore). 2017;96(11):e6326. doi:10.1097/MD.0000000000006326 28296752 PMC5369907

[28] Erickson SE, Vasilevskis EE, Kuzniewicz MW, . The effect of race and ethnicity on outcomes among patients in the intensive care unit: a comprehensive study involving socioeconomic status and resuscitation preferences. Crit Care Med. 2011;39(3):429-435. doi:10.1097/CCM.0b013e318206b3af 21187746 PMC3638889

[29] Joseph NP, Reid NJ, Som A, . Racial and ethnic disparities in disease severity on admission chest radiographs among patients admitted with confirmed coronavirus disease 2019: a retrospective cohort study. Radiology. 2020;297(3):E303-E312. doi:10.1148/radiol.2020202602 32673191 PMC7370353

[30] Hayes SL, Riley P, Radley DC, McCarthy D. Reducing racial and ethnic disparities in access to care: has the Affordable Care Act made a difference? The Commonwealth Fund. Published August 24, 2017. Accessed February 12, 2021. https://www.commonwealthfund.org/publications/issue-briefs/2017/aug/reducing-racial-and-ethnic-disparities-access-care-has 28836751

[31] Schaeffer K. The most common age among Whites in U.S. is 58—more than double that of racial and ethnic minorities. Published July 30, 2019. Accessed January 22, 2021. https://www.pewresearch.org/fact-tank/2019/07/30/most-common-age-among-us-racial-ethnic-groups/

[32] Artiga S, Rae M, Pham O, Hamel L, Muñana C. COVID-19 risks and impacts among health care workers by race/ethnicity. Published November 11, 2020. Accessed January 22, 2021. https://www.kff.org/racial-equity-and-health-policy/issue-brief/covid-19-risks-impacts-health-care-workers-race-ethnicity/

[33] Kaplan JB, Bennett T. Use of race and ethnicity in biomedical publication. JAMA. 2003;289(20):2709-2716. doi:10.1001/jama.289.20.2709 12771118

