# Supplementary material for: Assessment of Disparities Associated With a Crisis Standards of Care Resource Allocation Algorithm for Patients in 2 US Hospitals During the COVID-19 Pandemic
Source: JAMA Netw Open. 2021 Mar 19;4(3):e214149. doi: 10.1001/jamanetworkopen.2021.4149 (PMC7980099; doi:10.1001/jamanetworkopen.2021.4149)
Supplement: Supplement. — eFigure 1. Scoring How-To Guide eFigure 2. Study Flow Diagram eTable 1. Comorbidities Included in Triage Score eTable 2. Baseline Characteristics of Cohort by Minimum Priority Group eTable 3. Adjusted Association of Race and Ethnicity with Maximum and Minimum Priority Groups eTable 4. Adjusted Association of Race and Ethnicity with Maximum and Minimum Sequential Organ Failure Assessment Scores eTable 5. Adjusted Association of Race and Ethnicity with Maximum and Minimum Sequential Organ Failure Assessment Points eTable 6. Adjusted Association of Race and Ethnicity with Maximum and Minimum Priority Scores Including Patients with Missing Covariate Information eTable 7. Adjusted Association of Race and Ethnicity with Maximum and Minimum Priority Scores Without Median Income as a Model Covariate eTable 8. Adjusted Association of Race and Ethnicity with Maximum and Minimum Priority Scores Without Median Income or Primary Insurance as Model Covariates eFigure 3. Comparison of Relative Triage Priority Based on Minimum Points With and Without Inclusion of Longer-Term Mortality [file jamanetwopen-e214149-s001.pdf]

Supplemental Online Content

Gershengorn HB, Holt GE, Rezk A, et al. Assessment of disparities associated with a crisis standards of care resource allocation algorithm for patients in 2 US hospitals during the COVID-19 pandemic. *JAMA Netw Open*. 2021;4(3):e214149. doi:10.1001/jamanetworkopen.2021.4149

eFigure 1. Scoring How-To Guide

eFigure 2. Study Flow Diagram

eTable 1. Comorbidities Included in Triage Score

eTable 2. Baseline Characteristics of Cohort by Minimum Priority Group

eTable 3. Adjusted Association of Race and Ethnicity with Maximum and Minimum Priority Groups

eTable 4. Adjusted Association of Race and Ethnicity with Maximum and Minimum Sequential Organ Failure Assessment Scores

eTable 5. Adjusted Association of Race and Ethnicity with Maximum and Minimum Sequential Organ Failure Assessment Points

eTable 6. Adjusted Association of Race and Ethnicity with Maximum and Minimum Priority Scores Including Patients with Missing Covariate Information

eTable 7. Adjusted Association of Race and Ethnicity with Maximum and Minimum Priority Scores Without Median Income as a Model Covariate

eTable 8. Adjusted Association of Race and Ethnicity with Maximum and Minimum Priority Scores Without Median Income or Primary Insurance as Model Covariates

eFigure 3. Comparison of Relative Triage Priority Based on Minimum Points With and Without Inclusion of Longer-Term Mortality

This supplemental material has been provided by the authors to give readers additional information about their work.

© 2021 Gershengorn HB et al. *JAMA Network Open.*

eFigure 1. Scoring How-To Guide[a]

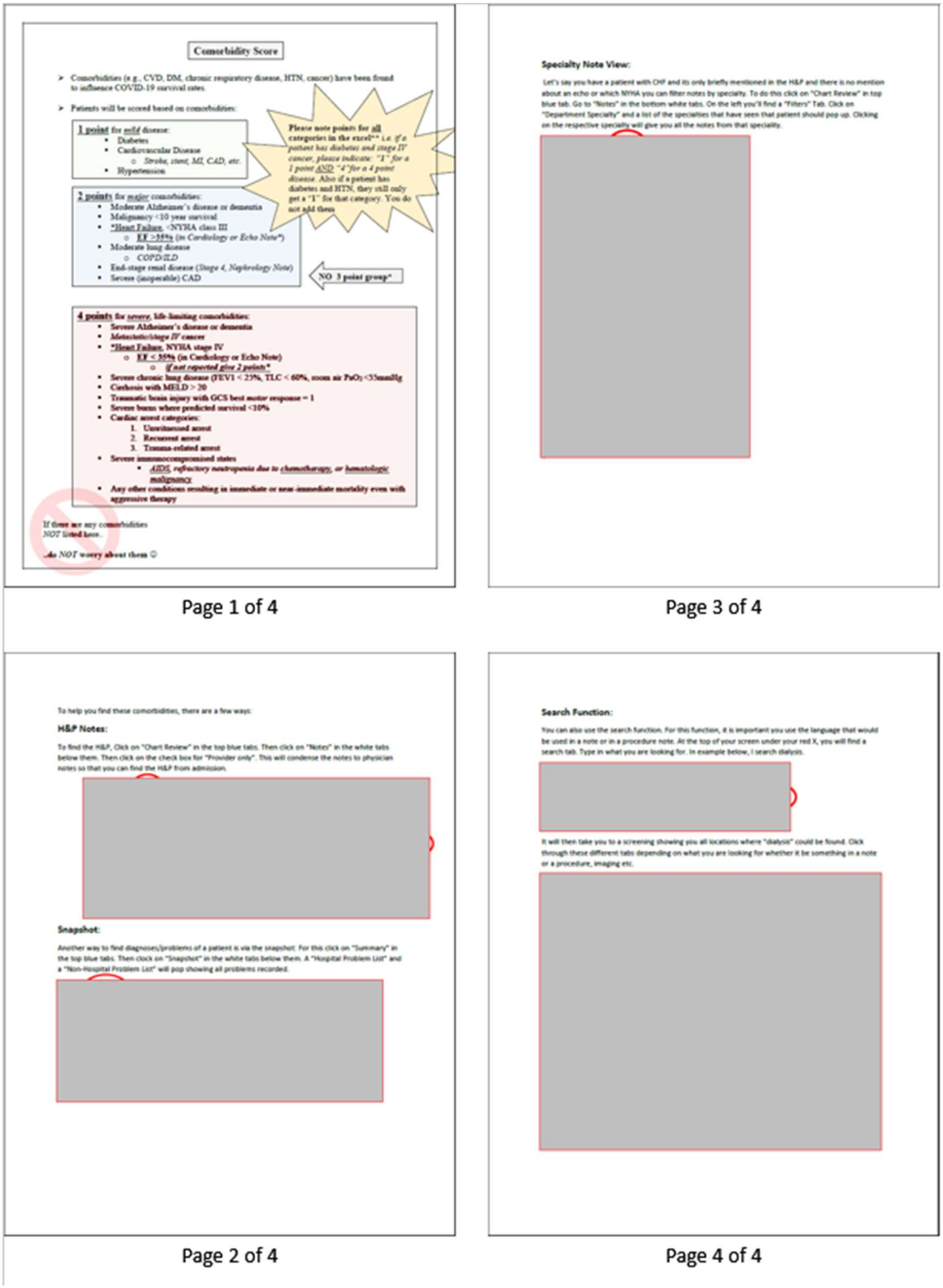

Comorbidity Score

- Comorbidities (e.g., CVD, DM, chronic respiratory disease, HTN, cancer) have been found to influence COVID-19 survival rates.
- Patients will be scored based on comorbidities:

1 point for *mild* disease:
- Diabetes
- Cardiovascular Disease
  - *Stroke, stent, MI, CAD, etc.*
- Hypertension

2 points for *major* comorbidities:
- Moderate Alzheimer's disease or dementia
- Malignancy <10 year survival
- *Heart Failure, <NYHA class III
  - EF >35% (*in Cardiology or Echo Note**)
- Moderate lung disease
  - *COPD/ILD*
- End-stage renal disease (*Stage 4, Nephrology Note*)
- Severe (inoperable) CAD

Please note points for all categories in the excel** *i.e. if a patient has diabetes and stage IV cancer, please indicate: "1" for a 1 point AND "4" for a 4 point disease.* Also if a patient has diabetes and HTN, they still only get a "1" for that category. You do not add them

NO 3 point group*

4 points for *severe*, life-limiting comorbidities:
- Severe Alzheimer's disease or dementia
- *Metastatic/stage IV cancer*
- *Heart Failure, NYHA stage IV
  - EF < 35% (in Cardiology or Echo Note)
    - *If not reported give 2 points**
- Severe chronic lung disease (FEV1 < 25%, TLC < 60%, room air PaO_2 <55mmHg
- Cirrhosis with MELD > 20
- Traumatic brain injury with GCS best motor response = 1
- Severe burns where predicted survival <10%
- Cardiac arrest categories:
  1. Unwitnessed arrest
  2. Recurrent arrest
  3. Trauma-related arrest
- Severe immunocompromised states
  - *AIDS, refractory neutropenia due to chemotherapy, or hematologic malignancy*
- Any other conditions resulting in immediate or near-immediate mortality even with aggressive therapy

If there are any comorbidities *NOT* listed here…

…do *NOT* worry about them ☺

Page 1 of 4

Specialty Note View:

Let's say you have a patient with CHF and its only briefly mentioned in the H&P and there is no mention about an echo or which NYHA you can filter notes by specialty. To do this click on "Chart Review" in top blue tab. Go to "Notes" in the bottom white tabs. On the left you'll find a "Filters" Tab. Click on "Department Specialty" and a list of the specialties that have seen that patient should pop up. Clicking on the respective specialty will give you all the notes from that specialty.

Page 3 of 4

To help you find these comorbidities, there are a few ways:

H&P Notes:

To find the H&P, Click on "Chart Review" in the top blue tabs. Then click on "Notes" in the white tabs below them. Then click on the check box for "Provider only". This will condense the notes to physician notes so that you can find the H&P from admission.

Snapshot:

Another way to find diagnoses/problems of a patient is via the snapshot. For this click on "Summary" in the top blue tabs. Then clock on "Snapshot" in the white tabs below them. A "Hospital Problem List" and a "Non-Hospital Problem List" will pop showing all problems recorded.

Page 2 of 4

Search Function:

You can also use the search function. For this function, it is important you use the language that would be used in a note or in a procedure note. At the top of your screen under your red X, you will find a search tab. Type in what you are looking for. In example below, I search dialysis.

It will then take you to a screening showing you all locations where "dialysis" could be found. Click through these different tabs depending on what you are looking for whether it be something in a note or a procedure, imaging etc.

Page 4 of 4

a 1-point for "mild disease" was used to collect and code data for the comorbidities known to affect short-term recovery which would be used as tie-breakers; gray boxes overlay screenshots of the electronic medical record demonstrating where to find needed data

© 2021 Gershengorn HB et al. *JAMA Network Open.*

eFigure 2. Study Flow Diagram

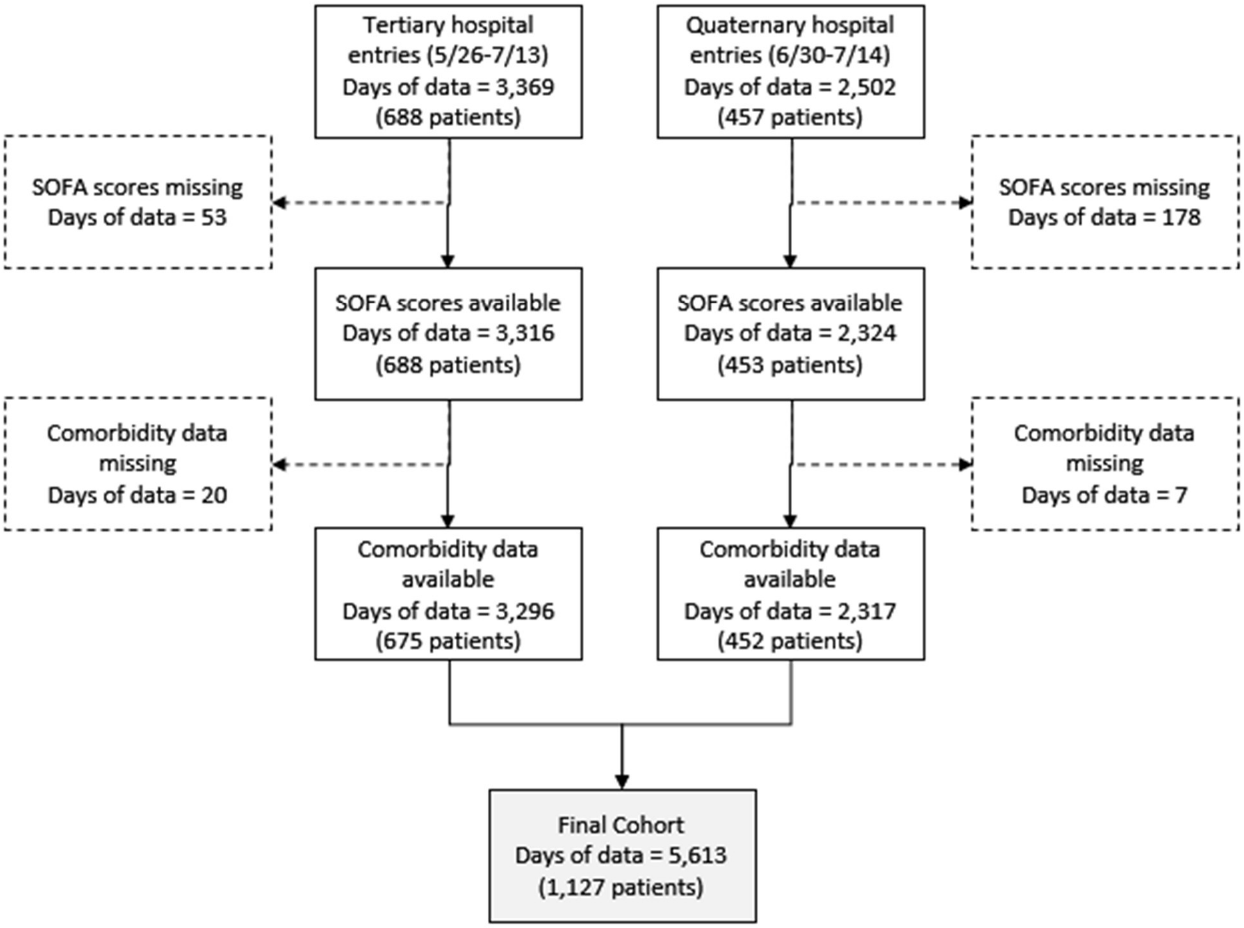

SOFA: Sequential Organ Failure Assessment

© 2021 Gershengorn HB et al. *JAMA Network Open.*

eTable 1. Comorbidities Included in Triage Score

**4-point Comorbidities**
• Severe Alzheimer's Disease or dementia
• Metastatic/stage IV cancer
• Heart failure (NYHA class IV or ejection fraction <35%)
• Severe lung disease (FEV1<25%, TLC<60%, or PaO2 on room air <55mmHg)
• Cirrhosis with MELD >20
• Traumatic brain injury with best GCS motor response = 1
• Severe burns where predicted survival <10%
• Cardiac arrest that is either unwitnessed, recurrent, or following trauma[a]
• Severe immunocompromised state (AIDS, refractory neutropenia due to chemotherapy, or hematologic malignancy)
**2-point Comorbidities**
• Moderate Alzheimer's Disease or dementia
• Malignancy with expected <10-year survival
• Heart failure (NYHA class III; ejection fraction ≥35% or not documented/available)
• Moderate lung disease (COPD or ILD not meeting "severe" criteria above)
• Severe or end-stage renal disease (stages 4 & 5)
• Coronary artery disease that is not able to be intervened on (via stent or surgery)
**Tie-Breaker Comorbidities**
• Diabetes mellitus
• Cardiovascular disease other than that above (stroke, stent, MI, CAD)
• Hypertension

AIDS: acquired immunodeficiency syndrome; CAD: coronary artery disease; COPD: chronic obstructive pulmonary disease; FEV1: forced expiratory volume in 1 second; GCS: Glasgow coma scale; ILD: interstitial lung disease; MELD: model for end-stage liver disease; MI: myocardial infarction; NYHA: New York Heart Association; PaO2: partial pressure of oxygen in the arterial blood; TLC: total lung capacity

a not actually "comorbidities" but, rather, current conditions likely to impact long-term survival

© 2021 Gershengorn HB et al. *JAMA Network Open.*

eTable 2. Baseline Characteristics of Cohort by Minimum Priority Group

	Full Cohort N (%)	Priority Group 1 N (%)	Priority Group 2 N (%)	Priority Group 3 N (%)	p-value (All Groups)	p-value (Group 3 vs 1)
# of patients, N (row %)	1127 (100.0)	865 (76.8)	232 (20.6)	30 (2.7)		
# of days of data per patient, median (IQR)	3 (2,7)	3 (2,6)	3 (2,7)	3 (2,8)	0.94	0.77
Age[a], median (IQR)	62.7 (51.7,73.7)	61.8 (50.4,72.9)	65.8 (55.8,75.0)	71.5 (57.8,79.0)	0.001	0.020
Comorbidities[b]						
Reduce 5-year survival	408 (36.2)	283 (32.7)	109 (47.0)	16 (53.3)	<0.001	0.019
Reduce 1-year survival	217 (19.3)	0 (0.0)	190 (81.9)	27 (90.0)	<0.001	<0.001
Race					0.31	0.68
White	711 (63.1)	543 (62.8)	147 (63.4)	21 (70.0)		
Black	323 (28.7)	255 (29.5)	61 (26.3)	7 (23.3)		
Asian	8 (0.7)	6 (0.7)	2 (0.9)	0 (0.0)		
Multi-race	31 (2.8)	27 (3.1)	4 (1.7)	0 (0.0)		
Unknown	54 (4.8)	34 (3.9)	18 (7.8)	2 (6.7)		
Ethnicity					0.50	0.45
Non-Hispanic	480 (42.6)	359 (41.5)	106 (45.7)	15 (50.0)		
Hispanic	611 (54.2)	479 (55.4)	117 (50.4)	15 (50.0)		
Unknown	36 (3.2)	27 (3.1)	9 (3.9)	0 (0.0)		
Sex					0.45	0.17
Male	607 (53.9)	464 (53.6)	125 (53.9)	19 (63.3)		
Female	509 (45.2)	394 (45.5)	104 (44.8)	10 (33.3)		
Neither/unknown	11 (1.0)	7 (0.8)	3 (1.3)	1 (3.3)		
Preferred language					0.68	0.90
English	591 (52.4)	443 (51.2)	131 (56.5)	17 (56.7)		
Spanish	489 (43.4)	383 (44.3)	94 (40.5)	12 (40.0)		
Other	35 (3.1)	30 (3.5)	4 (1.7)	1 (3.3)		
Unknown	12 (1.1)	9 (1.0)	3 (1.3)	0 (0.0)		
Primary insurance					<0.001	0.003
Medicare/Medicaid	360 (31.9)	254 (29.4)	88 (37.9)	18 (60.0)		
Commercial	589 (52.3)	451 (52.1)	127 (54.7)	11 (36.7)		
None	153 (13.6)	139 (16.1)	13 (5.6)	1 (3.3)		
Unknown	25 (2.2)	21 (2.4)	4 (1.7)	0 (0.0)		
Median annual income for zip code[c], *$1000					0.51	0.88
<$25	219 (19.4)	180 (20.8)	33 (14.2)	6 (20.0)		
$25-<$50	546 (48.4)	415 (48.0)	117 (50.4)	14 (46.7)		
$50-<$75	236 (20.9)	173 (20.0)	55 (23.7)	8 (26.7)		
$75+	71 (6.3)	53 (6.1)	17 (7.3)	1 (3.3)		
Unknown	55 (4.9)	44 (5.1)	10 (4.3)	1 (3.3)		
On COVID-19 unit[d]	690 (61.2)	585 (67.6)	93 (40.1)	12 (40.0)	<0.001	0.002

IQR: interquartile range

a Age used is that on June 1, 2020; data missing on 3 (0.3%) patients

b Presence of at least one comorbidity likely to reduce 1- and/or 5-year survival are both listed here; in assignment of points for priority scoring, patients with both comorbidities likely to reduce 1- and 5-year survival are only allocated points for the more severe (1-year) comorbidity burden

c Data missing on 55 (4.9%) patients

d COVID-19 status for each individual patient was not accessed; rather, this value indicates whether a patient was admitted to a ward serving COVID-19 patients as, in both hospitals, COVID-19 patients were segregated from non-COVID-19 patients by ward

© 2021 Gershengorn HB et al. *JAMA Network Open.*

eTable 3. Adjusted Association of Race and Ethnicity with Maximum and Minimum Priority Groups

	Maximum Priority Group		Minimum Priority Group	
	IRR (95% CI)	p-value	IRR (95% CI)	p-value
Race				
White	1		1	
Black	1.02 (0.87,1.21)	0.77	1.00 (0.85,1.19)	0.96
Asian	0.98 (0.53,1.80)	0.95	0.97 (0.51,1.83)	0.92
Multi-race	1.03 (0.73,1.45)	0.87	0.91 (0.62,1.34)	0.64
Ethnicity				
Non-Hispanic	1		1	
Hispanic	1.00 (0.85,1.18)	0.96	1.01 (0.85,1.20)	0.92
Sex				
Male	1		1	
Female	0.95 (0.86,1.06)	0.38	0.98 (0.88,1.10)	0.79
Preferred language				
English	1		1	
Spanish	0.97 (0.83,1.12)	0.66	0.98 (0.84,1.15)	0.83
Other	0.88 (0.64,1.21)	0.43	0.92 (0.66,1.28)	0.61
Median annual income for zip code, *$1000				
<$25	1		1	
$25-<$50	1.03 (0.90,1.18)	0.67	1.05 (0.91,1.22)	0.51
$50-<$75	0.97 (0.82,1.16)	0.75	1.01 (0.84,1.21)	0.91
$75+	0.92 (0.71,1.20)	0.54	0.99 (0.75,1.30)	0.94
Primary insurance				
Medicare/Medicaid	1		1	
Commercial	0.89 (0.79,1.00)	0.05	0.91 (0.81,1.03)	0.15
None	0.81 (0.67,0.98)	0.029	0.86 (0.71,1.04)	0.12
Age[a]	1.00 (1.00,1.01)	0.13	1.00 (1.00,1.01)	0.44
On COVID-19 unit	0.82 (0.71,0.95)	0.008	0.84 (0.72,0.98)	0.028
Quaternary hospital	0.98 (0.84,1.14)	0.82	1.00 (0.85,1.17)	1.00

CI: confidence interval; IRR: incident rate ratio

a age on June 1, 2020; modeled as a continuous variable

© 2021 Gershengorn HB et al. *JAMA Network Open.*

eTable 4. Adjusted Association of Race and Ethnicity with Maximum and Minimum Sequential Organ Failure Assessment Scores

	Maximum SOFA Score		Minimum SOFA Score	
	IRR (95% CI)	p-value	IRR (95% CI)	p-value
Race				
White	1		1	
Black	0.94 (0.85,1.04)	0.23	0.91 (0.80,1.03)	0.15
Asian	1.04 (0.74,1.46)	0.83	1.09 (0.71,1.68)	0.69
Multi-race	1.33 (1.12,1.59)	0.001	1.26 (0.97,1.64)	0.08
Ethnicity				
Non-Hispanic	1		1	
Hispanic	0.93 (0.84,1.02)	0.14	0.95 (0.84,1.08)	0.45
Sex				
Male	1		1	
Female	0.84 (0.79,0.90)	<0.001	0.79 (0.73,0.87)	<0.001
Preferred language				
English	1		1	
Spanish	0.94 (0.86,1.03)	0.20	0.88 (0.78,0.99)	0.039
Other	0.92 (0.76,1.11)	0.37	0.85 (0.66,1.10)	0.22
Median annual income for zip code, *$1000				
<$25	1		1	
$25-<$50	1.02 (0.94,1.11)	0.66	1.09 (0.98,1.23)	0.12
$50-<$75	0.92 (0.83,1.02)	0.11	0.93 (0.80,1.07)	0.29
$75+	0.72 (0.61,0.85)	<0.001	0.79 (0.63,0.98)	0.032
Primary insurance				
Medicare/Medicaid	1		1	
Commercial	0.92 (0.86,0.99)	0.018	0.89 (0.81,0.98)	0.015
None	0.84 (0.75,0.94)	0.003	0.78 (0.67,0.92)	0.003
Age[a]	1.01 (1.01,1.01)	<0.001	1.01 (1.01,1.01)	<0.001
On COVID-19 unit	0.83 (0.76,0.91)	<0.001	0.48 (0.42,0.54)	<0.001
Quaternary hospital	0.96 (0.88,1.05)	0.34	1.47 (1.28,1.69)	<0.001

CI: confidence interval; IRR: incident rate ratio; SOFA: Sequential Organ Failure Assessment

a age on June 1, 2020; modeled as a continuous variable

© 2021 Gershengorn HB et al. *JAMA Network Open.*

eTable 5. Adjusted Association of Race and Ethnicity with Maximum and Minimum Sequential Organ Failure Assessment Points

	Maximum SOFA Points		Minimum SOFA Points	
	IRR (95% CI)	p-value	IRR (95% CI)	p-value
Race				
White	1		1	
Black	0.93 (0.79,1.09)	0.37	0.95 (0.79,1.14)	0.58
Asian	1.14 (0.65,2.00)	0.65	1.21 (0.66,2.24)	0.54
Multi-race	1.23 (0.89,1.69)	0.20	1.02 (0.69,1.51)	0.92
Ethnicity				
Non-Hispanic	1		1	
Hispanic	0.97 (0.82,1.14)	0.69	1.01 (0.84,1.21)	0.93
Sex				
Male	1		1	
Female	0.88 (0.79,0.98)	0.016	0.95 (0.84,1.07)	0.36
Preferred language				
English	1		1	
Spanish	0.94 (0.81,1.09)	0.42	0.94 (0.80,1.11)	0.45
Other	0.87 (0.64,1.20)	0.41	0.90 (0.64,1.28)	0.56
Median annual income for zip code, *$1000				
<$25	1		1	
$25-<$50	1.01 (0.88,1.16)	0.92	1.05 (0.90,1.22)	0.56
$50-<$75	0.97 (0.81,1.15)	0.69	0.99 (0.82,1.20)	0.92
$75+	0.81 (0.62,1.07)	0.14	0.97 (0.72,1.30)	0.84
Primary insurance				
Medicare/Medicaid	1		1	
Commercial	0.96 (0.85,1.08)	0.48	0.98 (0.85,1.11)	0.72
None	0.90 (0.75,1.08)	0.25	0.96 (0.78,1.17)	0.66
Age[a]	1.00 (1.00,1.01)	0.041	1.00 (1.00,1.01)	0.46
On COVID-19 unit	0.87 (0.75,1.01)	0.06	0.89 (0.75,1.05)	0.16
Quaternary hospital	1.06 (0.91,1.23)	0.48	1.07 (0.91,1.27)	0.40

CI: confidence interval; IRR: incident rate ratio; SOFA: Sequential Organ Failure Assessment

a age on June 1, 2020; modeled as a continuous variable

© 2021 Gershengorn HB et al. *JAMA Network Open.*

eTable 6. Adjusted Association of Race and Ethnicity with Maximum and Minimum Priority Scores Including Patients with Missing Covariate Information[a]

	Maximum Priority Score		Minimum Priority Score	
	IRR (95% CI)	p-value	IRR (95% CI)	p-value
Race				
White	1		1	
Black	0.98 (0.88,1.09)	0.70	0.99 (0.88,1.11)	0.83
Asian	0.94 (0.62,1.43)	0.78	0.94 (0.61,1.46)	0.79
Multi-race	0.88 (0.69,1.12)	0.29	0.78 (0.60,1.01)	0.06
Unknown	1.26 (1.02,1.55)	0.029	1.28 (1.03,1.59)	0.028
Ethnicity				
Non-Hispanic	1		1	
Hispanic	0.97 (0.87,1.08)	0.53	0.98 (0.88,1.10)	0.78
Unknown	0.68 (0.51,0.92)	0.012	0.65 (0.47,0.88)	0.007
Sex				
Male	1		1	
Female	0.92 (0.85,0.99)	0.019	0.95 (0.88,1.02)	0.17
Neither/unknown	1.99 (1.20,3.31)	0.008	1.83 (1.06,3.18)	0.031
Preferred language				
English	1		1	
Spanish	0.94 (0.85,1.04)	0.22	0.94 (0.84,1.04)	0.25
Other	0.85 (0.68,1.06)	0.15	0.87 (0.68,1.09)	0.23
Unknown	0.76 (0.43,1.33)	0.33	0.77 (0.42,1.41)	0.39
Median annual income for zip code, *$1000				
<$25	1		1	
$25-<$50	1.07 (0.97,1.18)	0.17	1.09 (0.98,1.21)	0.10
$50-<$75	1.00 (0.88,1.13)	0.98	1.02 (0.90,1.16)	0.80
$75+	0.97 (0.82,1.16)	0.76	1.07 (0.89,1.28)	0.47
Unknown	0.96 (0.78,1.18)	0.67	0.98 (0.79,1.22)	0.87
Primary insurance				
Medicare/Medicaid	1		1	
Commercial	0.84 (0.78,0.91)	<0.001	0.84 (0.77,0.91)	<0.001
None	0.67 (0.58,0.77)	<0.001	0.66 (0.57,0.77)	<0.001
Unknown	0.66 (0.48,0.91)	0.011	0.73 (0.53,1.01)	0.06
Age[b]	1.01 (1.01,1.01)	<0.001	1.01 (1.00,1.01)	<0.001
On COVID-19 unit	0.71 (0.65,0.79)	<0.001	0.71 (0.63,0.78)	<0.001
Quaternary hospital	0.95 (0.85,1.05)	0.32	0.94 (0.84,1.05)	0.28

CI: confidence interval; IRR: incident rate ratio

a excluded 3 (0.3%) patients with missing information on age

b age on June 1, 2020; modeled as a continuous variable

© 2021 Gershengorn HB et al. *JAMA Network Open.*

eTable 7. Adjusted Association of Race and Ethnicity with Maximum and Minimum Priority Scores Without Median Income as a Model Covariate[a]

	Maximum Priority Score		Minimum Priority Score	
	IRR (95% CI)	p-value	IRR (95% CI)	p-value
Race				
White	1		1	
Black	1.01 (0.90,1.12)	0.91	1.01 (0.90,1.13)	0.89
Asian	0.95 (0.62,1.45)	0.82	0.96 (0.62,1.49)	0.84
Multi-race	0.93 (0.72,1.19)	0.56	0.81 (0.61,1.07)	0.13
Ethnicity				
Non-Hispanic	1		1	
Hispanic	0.99 (0.88,1.10)	0.82	1.00 (0.89,1.13)	1.00
Sex				
Male	1		1	
Female	0.93 (0.86,1.00)	0.06	0.97 (0.89,1.05)	0.43
Preferred language				
English	1		1	
Spanish	0.96 (0.87,1.07)	0.48	0.96 (0.86,1.07)	0.47
Other	0.87 (0.70,1.10)	0.24	0.89 (0.70,1.13)	0.33
Primary insurance				
Medicare/Medicaid	1		1	
Commercial	0.84 (0.77,0.91)	<0.001	0.84 (0.77,0.91)	<0.001
None	0.66 (0.57,0.76)	<0.001	0.66 (0.57,0.77)	<0.001
Age[b]	1.01 (1.00,1.01)	<0.001	1.01 (1.00,1.01)	<0.001
On COVID-19 unit	0.70 (0.63,0.78)	<0.001	0.69 (0.62,0.77)	<0.001
Quaternary hospital	0.98 (0.87,1.09)	0.67	0.98 (0.87,1.10)	0.71

CI: confidence interval; IRR: incident rate ratio

a excluded 3 (0.3%) patients with missing information on age

b age on June 1, 2020; modeled as a continuous variable

© 2021 Gershengorn HB et al. *JAMA Network Open.*

eTable 8. Adjusted Association of Race and Ethnicity with Maximum and Minimum Priority Scores Without Median Income or Primary Insurance as Model Covariates[a]

	Maximum Priority Score		Minimum Priority Score	
	IRR (95% CI)	p-value	IRR (95% CI)	p-value
Race				
White	1		1	
Black	1.01 (0.90,1.12)	0.91	1.01 (0.90,1.13)	0.88
Asian	0.92 (0.60,1.40)	0.70	0.92 (0.60,1.44)	0.73
Multi-race	0.94 (0.73,1.20)	0.61	0.81 (0.61,1.08)	0.15
Ethnicity				
Non-Hispanic	1		1	
Hispanic	0.96 (0.86,1.07)	0.47	0.97 (0.86,1.09)	0.64
Sex				
Male	1		1	
Female	0.94 (0.87,1.01)	0.08	0.97 (0.90,1.06)	0.53
Preferred language				
English	1		1	
Spanish	0.96 (0.86,1.07)	0.45	0.96 (0.86,1.07)	0.44
Other	0.89 (0.71,1.12)	0.33	0.91 (0.72,1.15)	0.43
Age[b]	1.01 (1.01,1.01)	<0.001	1.01 (1.01,1.01)	<0.001
On COVID-19 unit	0.69 (0.62,0.77)	<0.001	0.68 (0.61,0.76)	<0.001
Quaternary hospital	0.97 (0.87,1.08)	0.57	0.97 (0.86,1.09)	0.62

CI: confidence interval; IRR: incident rate ratio

a excluded 3 (0.3%) patients with missing information on age

b age on June 1, 2020; modeled as a continuous variable

© 2021 Gershengorn HB et al. *JAMA Network Open.*

eFigure 3. Comparison of Relative Triage Priority Based on Minimum Points With and Without Inclusion of Longer-Term Mortality[a]

A. All Patients

		Priority Group			
		group 1	group 2	group 3	total
SOFA points	group 1	75%	17%	0%	91%
	group 2	1%	3%	2%	5%
	group 3	1%	1%	1%	3%
	total	77%	21%	3%	

Including comorbidities results in:
3% ↑ and 19% ↓ relative priority

B. Race

White

		Priority Group			
		group 1	group 2	group 3	total
SOFA points	group 1	74%	17%	0%	91%
	group 2	1%	3%	2%	5%
	group 3	1%	1%	1%	3%
	total	76%	21%	3%	

Including comorbidities results in:
3% ↑ and 19% ↓ relative priority

Black

		Priority Group			
		group 1	group 2	group 3	total
SOFA points	group 1	77%	16%	0%	93%
	group 2	1%	2%	2%	5%
	group 3	1%	1%	1%	2%
	total	79%	19%	2%	

Including comorbidities results in:
3% ↑ and 18% ↓ relative priority

Asian

		Priority Group			
		group 1	group 2	group 3	total
SOFA points	group 1	75%	13%	0%	88%
	group 2	0%	0%	0%	0%
	group 3	0%	13%	0%	13%
	total	75%	25%	0%	

Including comorbidities results in:
13% ↑ and 13% ↓ relative priority

Multi-race

		Priority Group			
		group 1	group 2	group 3	total
SOFA points	group 1	81%	10%	0%	90%
	group 2	6%	3%	0%	10%
	group 3	0%	0%	0%	0%
		87%	13%	0%	

Including comorbidities results in:
6% ↑ and 10% ↓ relative priority

C. Ethnicity

Non-Hispanic

		Priority Group			
		group 1	group 2	group 3	total
SOFA points	group 1	72%	19%	0%	91%
	group 2	1%	2%	2%	5%
	group 3	1%	1%	1%	4%
	total	75%	22%	3%	

Including comorbidities results in:
3% ↑ and 21% ↓ relative priority

Hispanic

		Priority Group			
		group 1	group 2	group 3	total
SOFA points	group 1	77%	15%	0%	92%
	group 2	1%	3%	1%	5%
	group 3	1%	1%	1%	3%
	total	78%	19%	2%	

Including comorbidities results in:
3% ↑ and 16% ↓ relative priority

SOFA: Sequential Organ Failure Assessment
a SOFA points 3 and 4 combined into single group (group 3)

© 2021 Gershengorn HB et al. *JAMA Network Open.*
